# Intracranial Hemorrhage Following Oral Low-Dose Methotrexate After Multiple Toxicities Caused by High-Dose Methotrexate in Childhood Acute Lymphoblastic Leukemia

**DOI:** 10.3389/fphar.2019.01072

**Published:** 2019-09-19

**Authors:** Ning Xin, Zhou Fen, Cheng Li, Xiao Yan, Jin Runming

**Affiliations:** Department of Pediatrics, Union Hospital, Tongji Medical College, Huazhong University of Science and Technology, Wuhan, China

**Keywords:** intracranial hemorrhage, methotrexate, toxicity, neurotoxicity, acute lymphoblastic leukemia

## Abstract

An 11-year-old male patient with the deletion of *IKZF1* (Ikaros family zinc finger 1) and positive Breakpoint Cluster Region-C-Abelson oncogene 1(*BCR-ABL1*) acute lymphoblastic leukemia developed mucositis, gastrointestinal toxicity, hepatotoxicity, myelosuppression, and severe dermatologic toxicity during the first and second courses of high-dose methotrexate. The patient recovered completely after therapy. However, intracranial hemorrhage (ICH) occurred following oral methotrexate at a dose of 25 mg/m^2^ in maintenance treatment, and he had neurological sequelae including hemiplegic paralysis.

## Introduction

An 11-year-old male patient with an *IKZF1* (Ikaros family zinc finger 1) deletion and BCR-ABL1–positive acute lymphoblastic leukemia (ALL) developed elevated levels of serum alanine aminotransferase and cholecystitis with symptoms of vomiting, abdominal pain, and diarrhea during the first course of high-dose methotrexate. He was admitted for myelosuppression, oral mucositis, rashes, and liver dysfunction during the second treatment of high-dose methotrexate. Ultimately, intracranial hemorrhage (ICH) occurred following oral methotrexate at a dose of 25 mg/m^2^ in maintenance treatment, and computed tomography (CT) scan of his brain showed increased intensities in multiple cerebral lobes. He had neurological sequelae of hemiplegic paralysis after 6 months.

### Background

Methotrexate is an essential component of therapy for acute lymphoblastic leukemia (ALL). It has significant antileukemia effects and offers the advantage of targeting extramedullary leukemia. There are multiple side effects, such as myelosuppression, mucositis, dermatologic toxicity, hepatotoxicity, and neurotoxicity, particularly when administered intravenously as high-dose methotrexate (HD-MTX), but these are not common with oral methotrexate.

In this study, we describe an ALL child who exhibited severe toxic events during HD-MTX therapy and intracranial hemorrhage (ICH) following oral methotrexate at a low dose.

### Case Presentation

An 11-year-old male patient was diagnosed with acute B-cell-progenitor ALL with the *BCR-ABL1*(Breakpoint Cluster Region-C-Abelson oncogene 1) fusion gene and *BCR-ABL* (Ikaros family zinc finger 1) deletion. Then, he received risk-directed chemotherapy according to the Chinese Children’s Cancer Group ALL 2015 (CCCG-ALL-2015) protocol (Cai et al., 2019). There was no evidence of central nervous system (CNS) infiltration, and early assessment of minimal residual disease was negative. Methylenetetrahydrofolate reductase (MTHFR) genotyping for *MTHFR C677T* and *A1298C* polymorphisms revealed *677CC* (the wild type) and *1298AC* genotypes in this patient, which suggested low risk of MTX toxicity.

**Figure 1:**
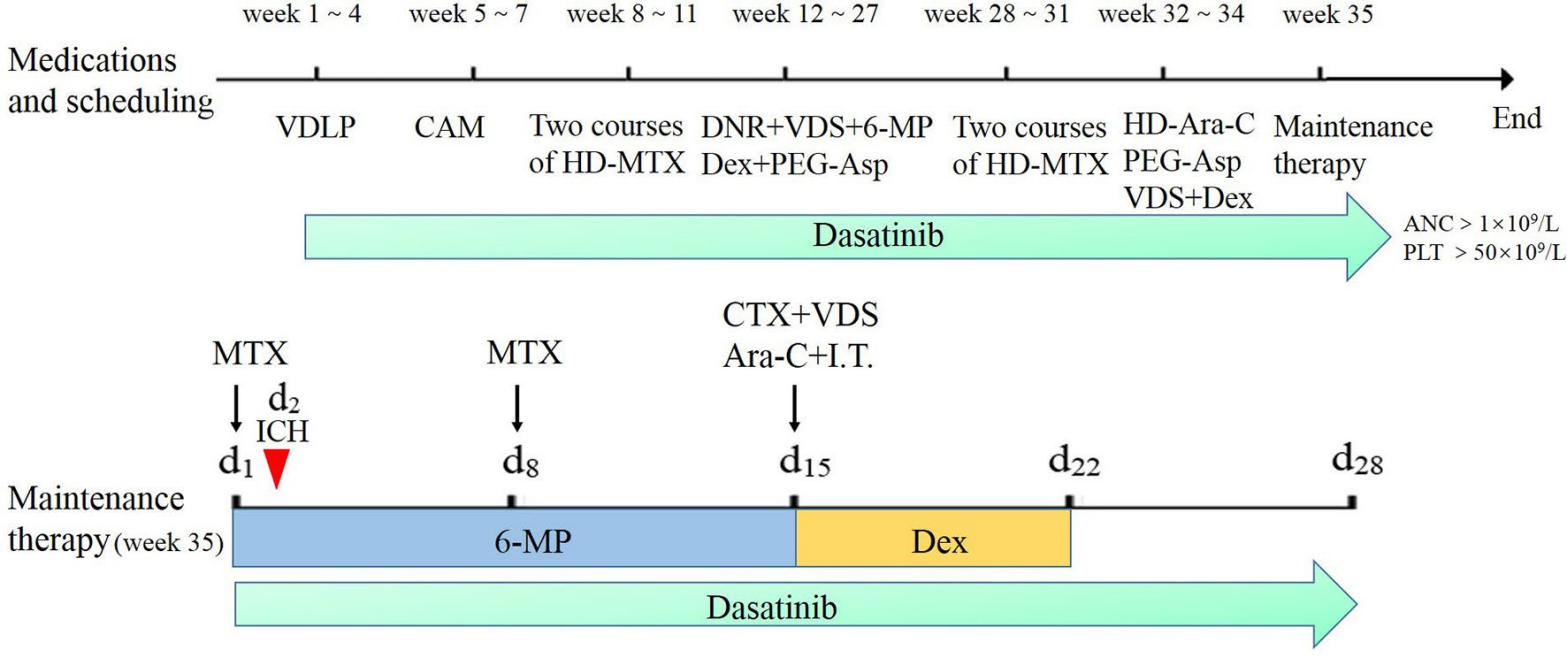
The chemotherapy process of the patient based on the Chinese Children’s Cancer Group ALL 2015 (CCCG-2015-ALL) protocol. The red arrow represents the time of developing intracranial hemorrhage at the start of maintenance therapy. Dasatinib was given when neutrophil count was more than 1×10^9^/L with platelet count more than 50×10^9^/L. Maintenance therapy methotrexate (MTX) was given respectively at day 1 and day 8 with oral daily mercaptopurine (6-MP) for 14 days; cyclophosphamide (CTX), vindesine (VDS), cytarabine (Ara-C), and intrathecal therapy (I.T.) were given at day 15 monthly with oral dexamethasone (Dex) from day 15 to day 22.

The process of chemotherapy is shown in **Figure 1**. He received induction treatment including VDLP, which consisted of vindesine (VDS), daunorubicin (DNR), L-asparaginase and prednisone, and CAM with its component drugs involving cyclophosphamide (CTX), cytarabine (Ara-C), and mercaptopurine (6-MP). Then, the two courses of HD-MTX therapy were given, each followed by multiple toxicities. Because of severe toxicity of MTX, he then received continuous therapy of five courses of interval treatment including DNR, VDS, 6-MP, dexamethasone (Dex) and pegaspargase (PEG-Asp), followed by another two courses of HD-MTX. After reinduction therapy including high-dose cytarabine (HD-Ara-C), the patient started to receive maintenance therapy. During the whole chemotherapy, dasatinib was administrated. The maintenance therapy included oral daily 6-MP (50mg/m^2^/day) and weekly MTX (25 mg/m^2^/day), along with intravenous (IV) CTX (300 mg/m^2^/day), VDS (3 mg/m^2^/day), Ara-C (300 mg/m^2^/day), and Dex (8 mg/m^2^/day). These latter four drugs and intrathecal therapy (I.T.) were given every 4 weeks. However, he developed ICH on the first 2 days of maintenance therapy.

**Figure 2:**
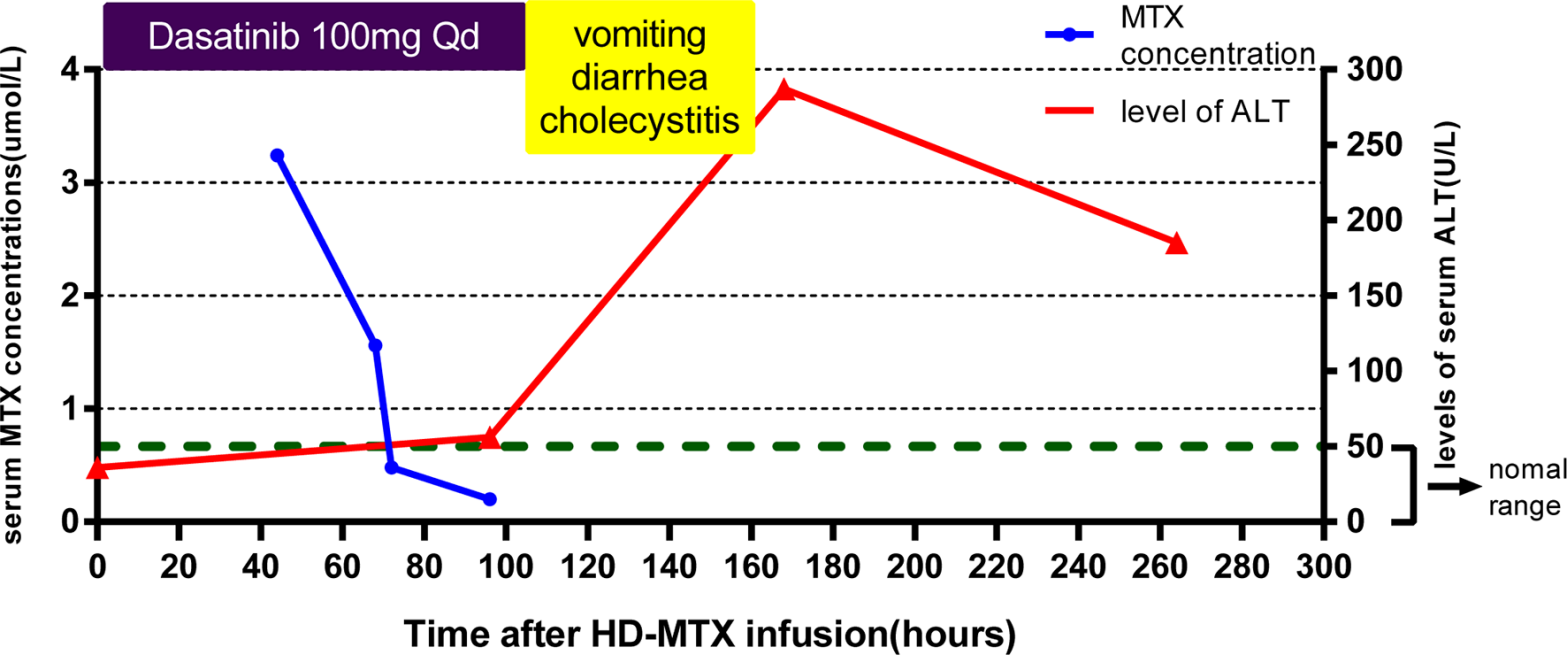
Clinical presentation of the patient during the first course of high-dose methotrexate (HD-MTX). MTX concentrations were monitored closely at 44, 68, 72, and 96 h after MTX infusion until the target level was reached. The patient developed vomiting, diarrhea, and cholecystitis at day 4 after infusion. The highest level of serum alanine aminotransferase (ALT) occurred at day 7. Dasatinib was withdrawn at the onset of vomiting and diarrhea.

**Figure 3:**
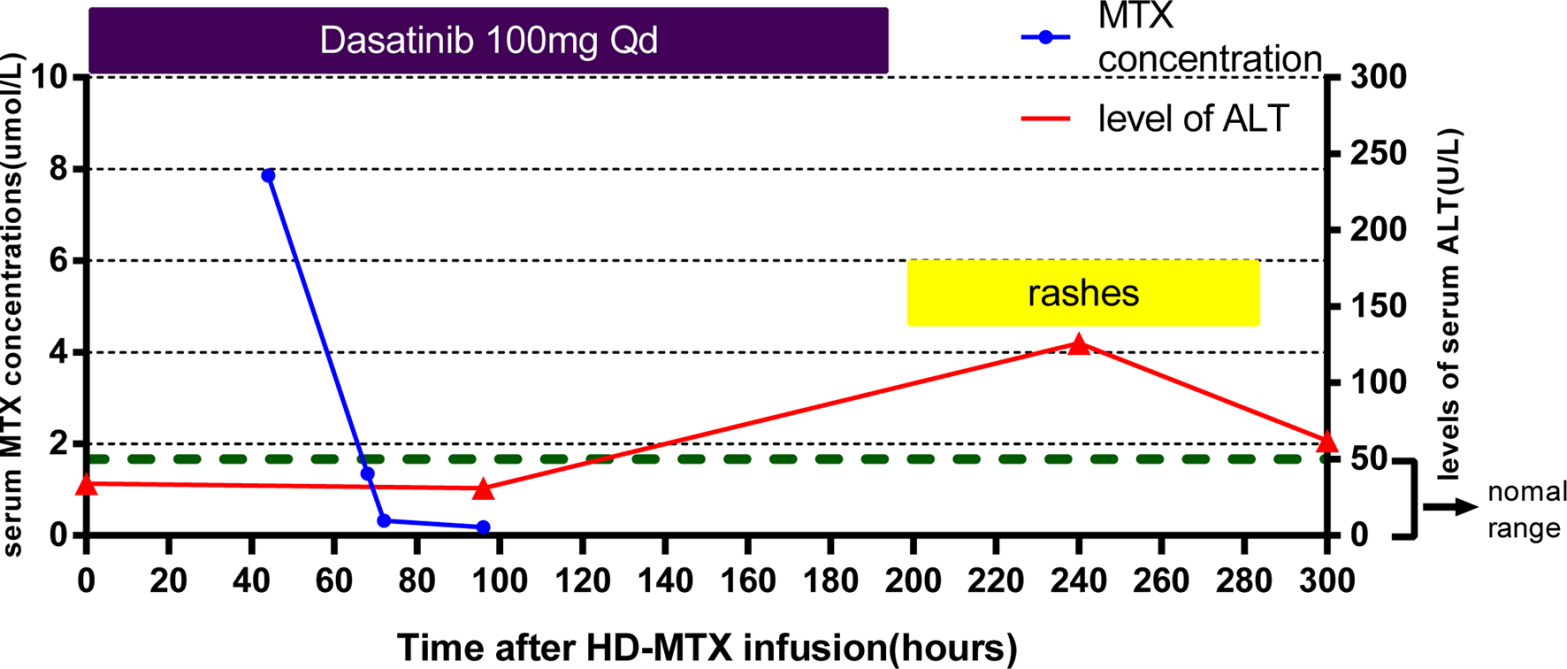
Clinical presentation of the patient during the second course of HD-MTX. MTX concentrations were monitored closely at 44, 68, 72, and 96 h after MTX infusion until the target level was reached. Skin toxicity appeared at day 8, with the highest level of serum ALT appearing day 10 after HD-MTX infusion. Dasatinib was withdrawn at the onset of rashes.

**Figure 4:**
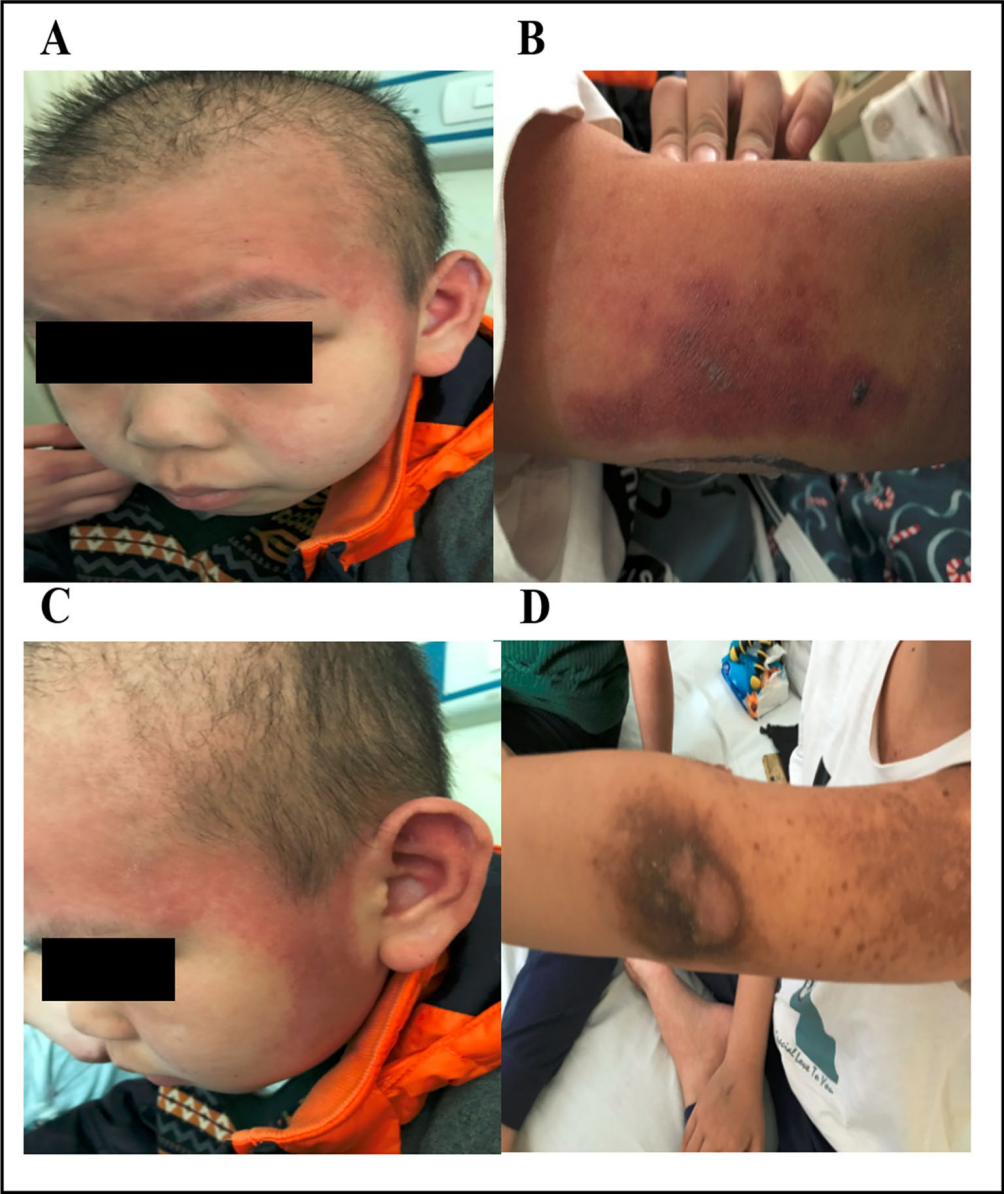
Dermatologic toxicity after treatment with HD-MTX. The patient developed skin erosions that were distributed over his face and arms **(A** and **B)**. Fading rashes and hyperpigmentation appeared after treatment **(C** and **D)**. Written informed consent for the publication of this image was obtained from the parents of the patient.

The dose of the first course of HD-MTX was 5 g/m^2^, and the MTX serum level reached 3.24 µmol/L at 44 h after the start of MTX infusion. Within 1 week after completing HD-MTX chemotherapy, the patient developed cholecystitis with symptoms of vomiting, abdominal pain, and diarrhea. The laboratory results showed elevated levels of serum alanine aminotransferase (ALT) and bilirubin. The patient recovered completely after supportive care, hydration, and glutathione. The clinical presentation is shown in **Figure 2**. According to the protocol, the dose of the second course of HD-MTX was reduced to 4 g/m^2^. The 44 h MTX serum level was 7.26 µmol/L, and leucovorin rescue was administered. He was admitted again for myelosuppression, oral mucositis, rashes, and liver dysfunction. **Figure 3** shows the clinical presentation during his second course. The most serious side effect was mucocutaneous toxicity, featuring dental ulcers and itchy erythema distributed over his face, arms, and trunk (**Figure 4**). Except for dasatinib, no other contributing factors (e.g., renal dysfunction or interacting drugs) have been identified. Dexamethasone, a histamine antagonist, hydration, and alkalization were administered, and he recovered. Leucovorin was started at 42 h after HD-MTX and administrated as IV infusion every 6 h, until the plasma MTX concentration was less than 0.2 μmol/L. The detailed dosage is shown in **Supplement 1**. The clinical laboratory investigations during the two courses of HD-MTX are shown in **Table 1**. This patient represented elevated levels of ALT and bilirubin, but normal renal function after MTX infusion, and serum MTX concentrations were examined during HD-MTX therapy.

**Table 1:** Clinical laboratory results during the two courses of HD-MTX.

Clinical parameters during HD-MTX therapy	Hours after the start of HD-MTX infusion								
	0 h	44 h	68 h	72 h	96 h	7 d	8 d	9 d	12 d
**First course of HD-MTX**									
MTX concentration (μmol/L)	–	3.24	1.56	0.48	0.20				–
ALT (U/L)	N	–	–	–	56.0 ↑	287.0↑	–	185.0↑	–
AST (U/L)	N	–	–	–	N	202.0↑	–	99.0 ↑	–
Total bilirubin (μmol/L)	N	–	–	–	N	27.1 ↑	–	75.3 ↑	–
Direct bilirubin (μmol/L)	N	–	–	–	N	12.3 ↑	–	57.1 ↑	–
Serum creatinine (mmol/L)	N	–	–	–	N	N	–	N	–
BUN (μmol/L)	N	–	–	–	N	N	–	N	–
**Second course of HD-MTX**									
MTX concentration (μmol/L)	–	7.86	1.35	0.32	0.18				–
ALT (U/L)	N	–	–	–	N	–	126.0↑	–	62.0 ↑
AST (U/L)	N	–	–	–	N	–	78.0 ↑	–	N
Total bilirubin (μmol/L)	N	–	–	–	N	–	21.3 ↑	–	N
Direct bilirubin (μmol/L)	N	–	–	–	N	–	13.0 ↑	–	N
Serum creatinine (mmol/L)	N	–	–	–	N	–	52.0 ↑	–	N
BUN (泐μmol/L)	N	–	–	–	N	–	N	–	N

HD-MTX, high-dose methotrexate; ALT, alanine aminotransferase; AST, Aspartate aminotransferase; BUN, blood urea nitrogen; N, normal range.

**Figure 5:**
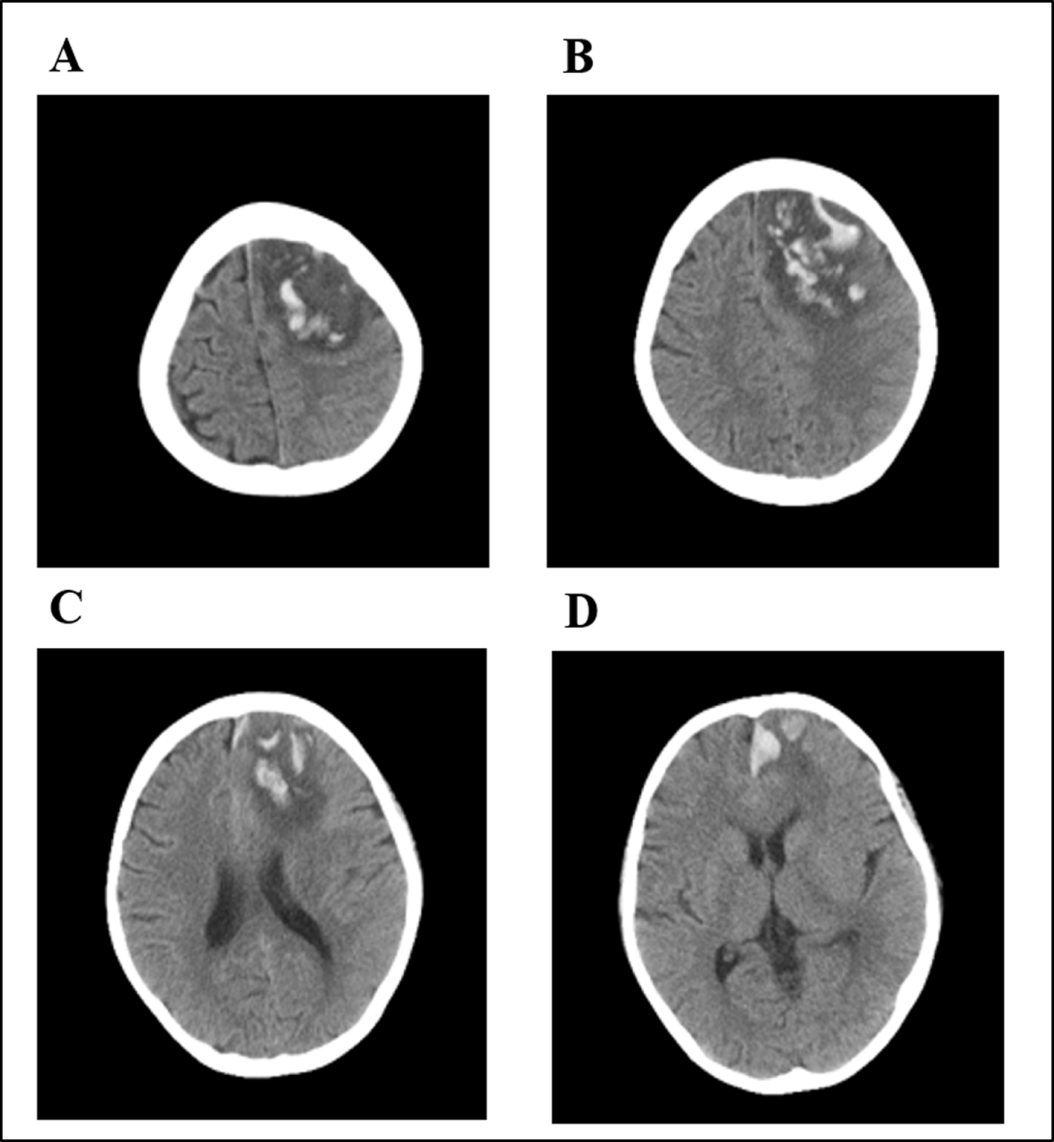
Brain computed tomography (CT) of the patient after oral low-dose MTX. Multiple hematomas were shown involving the left frontal **(A)**, parietal **(B)**, and temporal lobes **(C)** and the centrum semiovale **(D)**.

The subsequent two HD-MTX courses were safely administered with a reduced MTX dosage of 2 g/m^2^ and prolonged hydration and alkalization to prevent toxic events. However, during maintenance chemotherapy, the patient came to the hospital complaining of headache after oral methotrexate. In the following 24 h, he progressively developed repetitive seizures and loss of consciousness. His blood pressure was normal. The laboratory examinations showed no abnormalities in complete blood count, liver function, kidney function, blood glucose level, serum electrolytes, or coagulation function. A CT scan of his brain showed increased densities that were associated with multiple hematomas in the left frontal, parietal, and temporal lobes and the centrum semiovale (**Figure 5**). CT cerebral angiography was performed to rule out intracranial aneurysm. Ultimately, the patient gradually recovered after discontinuation of oral MTX and supportive care. Thereafter, during 6 months of follow-up, the patient received only tyrosine kinase inhibitor (TKI) therapy and had neurological sequelae of hemiplegic paralysis.

Given the temporal relationship between oral MTX and the development and resolution of the ICH, the Naranjo scale in **Table 2** was applied to assess the probability scale of adverse drug reaction (ADR). This patient could be scored in the “probable” range, because this adverse event (i) followed a reasonable temporal sequence after a drug, (ii) followed the improvement of this adverse event after discontinuation of a drug, and (iii) could not be reasonably explained by other compounding drugs or the known characteristics of the patient’s state. Therefore, ICH was probably associated with oral MTX according to the Naranjo scale (score = 5).

**Table 2:** Adverse drug reaction (ADR) probability scale.

To assess the adverse drug reaction, please answer the following questionnaire and give the pertinent score.			
	Yes	No	Do not know
1. Are there previous conclusive reports on this reaction?	+1	0√	0
2. Did the adverse event appear after the suspected drug was administered?	+2√	−1	0
3. Did the adverse reaction improve when the drug was discontinued or a specific antagonist was administered?	+1√	0	0
4. Did the adverse reaction reappear when the drug was readministered?	+2	−1	0√
5. Are there alternative causes (other than the drug) that could on their own have caused the reaction?	−1	+2√	0
6. Did the reaction reappear when a placebo was given?	−1	+1	0√
7. Was the drug detected in the blood (or other fluids) in concentrations known to be toxic?	+1	0	0√
8. Was the reaction more severe when the dose was increased, or less severe when the dose was decreased?	+1	0	0√
9. Did the patient have a similar reaction to the same or similar drugs in any previous exposure?	+1	0√	0
10. Was the adverse event confirmed by any objective evidence?	+1	0√	0

Total score as follows: definite ≥9; probable 5 to 8; possible 1 to 4; doubtful ≤0.

## Discussion

Methotrexate can indeed cause various side effects. The likelihood and severity of these toxic events tend to increase with the dose. Neurotoxicity is more commonly seen in patients receiving intrathecal or IV HD-MTX treatment. A neurological adverse event can be attributed to MTX if CNS symptoms (e.g., seizure, stroke, behavioral changes, and aphasia) occur within 2 weeks after receiving MTX and other identifiable causes are reasonably ruled out. The most common neurological events are transient ischemic attack, transient stroke-like symptoms, and encephalopathy (Rubnitz et al., 1998; Bhojwani et al., 2014; Millan et al., 2018). This toxicity usually subsides spontaneously and rarely leads to long-term sequelae (Rubnitz et al., 1998; Bhojwani et al., 2014). There are a few case reports showing that ICH occurred after intrathecal methotrexate therapy in adult and childhood ALL (Hentschke et al., 1999; Colosimo et al., 2000; Chia and Bazargan, 2015; Purkait et al., 2016; Ureshino et al., 2016).

ICH has been a known complication of I.T. methotrexate but has never been reported, since it has been used for decades in the treatment of autoimmune diseases such as rheumatoid arthritis. The patient in our report presented with CNS symptoms within 24 h after oral low-dose methotrexate during maintenance chemotherapy and suffered from ICH with hemiplegic paralysis. He did not receive I.T. or any other co-medications except dasatinib and 6-MP before and during the development of symptoms; he recovered gradually after discontinuation of MTX and supportive care; cerebrovascular malformation and CNS infiltration were excluded; therefore, ICH was probably associated with oral MTX. Our report suggests that MTX-induced neurotoxicity can occur during different courses of chemotherapy. Oral low-dose methotrexate can possibly lead to life-threatening ICH.

This patient received reinduction therapy including triple I.T. and co-medication (HD-Ara+Dex+PEG-Asp+VDS) 21 days before the start of maintenance therapy. The most common drug that in hematologic malignancies leads to hemorrhagic complications is L-asparaginase. However, ICH following L-asparaginase is mostly due to the disturbances of coagulation function such as hypofibrinogenemia and prolonged plasma clotting times, and often occurs within 2–3 weeks during the early induction therapy based on clinical observation and some case reports (Cairo et al., 1980; Priest et al., 1980; Pardes-Chavanes et al., 2016). This patient received L-asparagine during reinduction therapy along with normal coagulation function. Also, thromboembolism is a main complication of delayed ADR associated with asparaginase and Dex treatment during reinduction therapy (Lejhancova-Tousovska et al., 2012). Therefore, it is less likely to consider ICH associated with asparaginase. Besides, ICH could also be diagnosed several days to more than 3 months post-I.T. methotrexate, and the presence of headache post-I.T. is one of the most important risks in developing ICH (Chia and Bazargan, 2015). This patient had been safely given I.T. according to the protocol and never had headache post-I.T. The Naranjo scale showed a very low possibility of this association. Another drug that could cause ICH is dasatinib for its myelosuppression to increase the risk of bleeding. However, there is no evidence of myelosuppression in this patient. In conclusion, methotrexate is believed to be the most possible association among these drugs in proximity of ICH.

Skin erosions in this patient were unusual and atypical because dermatologic toxicity is often related to epidermal necrosis and cutaneous ulceration (Kazlow et al., 2003; Chen et al., 2017; Ferrari et al., 2018). In this case, this patient’s rashes included itchy erythema with a symmetrical distribution over his face, arms, and trunk, and dexamethasone, a histamine antagonist, hydration, and alkalinization were effective treatments for the skin lesions. These rash characteristics are akin to those caused by allergic drug reactions to methotrexate. Allergic dermatitis was a possibility, but the abnormal timing and hyperpigmentation of the rashes and the patient’s prior allergic history and subsequent exposure to MTX made this unlikely, generating a diagnosis of exclusion.

The patient suffered from hepatotoxicity, gastrointestinal toxicity, myelosuppression, and dermatologic toxicity during treatment with HD-MTX, and ICH following oral methotrexate. To analyze the reason, the first consideration is the combination of MTX and dasatinib. Ramsey et al. (Ramsey et al., 2019) reported that patients receiving tyrosine kinase inhibitor (TKI) treatment exhibit slower MTX clearance. This delay was mediated by SLCO1B1, a transporter known to transport methotrexate (Trevino et al., 2009; Ramsey et al., 2012; Ramsey et al., 2013). MTX polyglutamates (MTX-PGs), which are the metabolites of MTX, can be retained in cells for months and can be hydrolyzed by glutamate hydrolase to produce MTX in plasma. The continuous intake of MTX can cause the accumulation of MTX-PGs inside the cells, and MTX-PG concentrations will increase and reach a stead -state after 4–5 weeks’ administration. It is reported that the effect and toxicity of MTX are associated with MTX-PGs. In our report, the toxicity during the second course of MTX is more serious than that during the first one. The ICH happened on the first day of maintenance treatment, but the patient received the last course of HD-MTX therapy 5 weeks prior. These may be explained by the MTX-PG accumulation. The second consideration is the alteration of *IKZF1*, which may increase the possibility of adverse events. *IKZF1* deletion is strongly associated with an increased risk of relapse and adverse events (Medeiros, 2009; van der Veer et al., 2013; Tran et al., 2018). Therefore, avoiding methotrexate-induced severe toxic effects in higher-risk patients undergoing TKI treatment and multiple toxicities is difficult.

In conclusion, MTX-related toxicity is associated with MTX dosage and administration, drug metabolic gene, and specific drug combinations. It is important for clinicians and pharmacists to be aware of the great inter-individual variability in MTX pharmacokinetics, efficacy, and toxicity.

## Ethics Statement

Written informed consent was obtained from the parents of this patient for the publication of this case report and accompanying images. The Medical Ethics Committee of Union Hospital affiliated with Tongji Medical College of Huazhong University of Science and Technology approved this study, and the institutional review board gave us the permission to publish this case report and accompanying images.

## Author Contributions

JR and XY approved our collection of this patient’s medical history. NX and ZF wrote the manuscript. CL helped collect the information during the follow-up period. ZF contributed to conceiving the report and edited the manuscript. All authors reviewed and approved the manuscript.

## Conflict of Interest Statement

The authors declare that the research was conducted in the absence of any commercial or financial relationships that could be construed as a potential conflict of interest.
