# Supplementary material for: Intracranial Hemorrhage Following Oral Low-Dose Methotrexate After Multiple Toxicities Caused by High-Dose Methotrexate in Childhood Acute Lymphoblastic Leukemia
Source: Front Pharmacol. 2019 Sep 19;10:1072. doi: 10.3389/fphar.2019.01072 (PMC6761274; doi:10.3389/fphar.2019.01072)
Supplement: Supplementary file 1 [file Table_1.docx]

Supplement 1

Rescue scheme:

| [MTX]μΜ(44~48h) | [MTX]μΜ(68~72h) | CF(each dose) |
| --- | --- | --- |
| ≤1.0 | ≥^※^DL and ≤0.4 | 15mg/m^2^ |
| 1.0≤[MTX]≤2.0 | 0.4≤[MTX]≤0.5 | 30mg/m^2^ |
| 2.0≤[MTX]≤3.0 | 0.5≤[MTX]≤0.6 | 45mg/m^2^ |
| 3.0≤[MTX]≤4.0 | 0.6≤[MTX]≤0.8 | 60mg/m^2^ |
| 4.0≤[MTX]≤5.0 | 0.8≤[MTX]≤1.0 | 75mg/m^2^ |
| 5.0≤[MTX]≤6.0 | 1.0≤[MTX]≤1.5 | 90mg/m^2^ |
| 6.0≤[MTX]≤7.0 | 1.5≤[MTX]≤2.0 | 100mg/m^2^ |
| 7.0≤[MTX]≤8.0 | 2.0≤[MTX]≤3.0 | 120mg/m^2^ |
| 8.0≤[MTX]≤9.0 | 3.0≤[MTX]≤4.0 | 140mg/m^2^ |
| 9.0≤[MTX]≤10.0 | 4.0≤[MTX]≤5.0 | 160mg/m^2^ |
| ＞10 | ＞5 | 200mg/m2+dialysis |
| Discontinue rescue when MTX reviewed every 24 h ＜^※^DL ^※^DL: detectable low limit | | |
